# Structure–property relation and relevance of beam theories for microtubules: a coupled molecular and continuum mechanics study

**DOI:** 10.1007/s10237-017-0964-9

**Published:** 2017-10-03

**Authors:** Si Li, Chengyuan Wang, Perumal Nithiarasu

**Affiliations:** Zienkiewicz Centre for Computational Engineering, College of Engineering, Swansea University, Bay Campus, Fabian Way, Swansea, Wales, SA1 8EN UK

**Keywords:** Microtubules, Molecular structure mechanics model, Inter-*PF* sliding, Euler beam, Timoshenko beam, Nonlocal effect

## Abstract

Quasi-one-dimensional microtubules (MTs) in cells enjoy high axial rigidity but large transverse flexibility due to the inter-protofilament (PF) sliding. This study aims to explore the structure–property relation for MTs and examine the relevance of the beam theories to their unique features. A molecular structural mechanics (MSM) model was used to identify the origin of the inter-PF sliding and its role in bending and vibration of MTs. The beam models were then fitted to the MSM to reveal how they cope with the distinct mechanical responses induced by the inter-PF sliding. Clear evidence showed that the inter-PF sliding is due to the soft inter-PF bonds and leads to the length-dependent bending stiffness. The Euler beam theory is found to adequately describe MT deformation when the inter-PF sliding is largely prohibited. Nevertheless, neither shear deformation nor the nonlocal effect considered in the ‘more accurate’ beam theories can fully capture the effect of the inter-PF sliding. This reflects the distinct deformation mechanisms between an MT and its equivalent continuous body.

## Introduction

Microtubules (MTs) are a structural element and primary organizer in the cytoskeleton of eukaryotic cells (Chretien and Fuller 2000). They form “tracks” on which motor proteins transport organelles and construct the spindle apparatus to facilitate cell division (Howard and Hyman 2003). They are also responsible for maintaining the shape and providing the rigidity of the cells. The mechanics of MTs (Felgner et al. 1996; Gao and Lei 2009; Gittes et al. 1993; Kikumoto et al. 2006; Li et al. 2006; Takasone et al. 2002; Tounsi et al. 2010; Valdman et al. 2012; Zhang and Wang 2017) has been studied extensively in the last two decades, where the length dependency of equivalent bending stiffness (*EI*)$_{\mathrm{eq}}$ was captured as a unique feature of MTs and interpreted primarily via the continuum mechanics models (CMMs).

The Euler beam (EB) model was the first one used for MTs (Dogterom and Yurke 1997; Gittes et al. 1993; Kurachi et al. 1995; Takasone et al. 2002; Venier et al. 1994; Vinckier et al. 1996; Wang et al. 2001). In 1993, Gittes et al. measured (*EI*)$_{\mathrm{eq}}$ for MTs by fitting it to experiments (Gittes et al. 1993). In 2002, using this technique Kis et al. (2002) first reported the length-dependence of (*EI*)$_{\mathrm{eq}}$ for MTs and attributed it to their low shear modulus *G*. This theory (Kis et al. 2002) was then used by Kasas et al. to study the effect of anisotropy on MTs via the finite element method (Kasas et al. 2004). Pampaloni et al. also employed the theory to understand the length-dependent (*EI*)$_{\mathrm{eq}}$ achieved experimentally (Pampaloni et al. 2006).

**Fig. 1:**
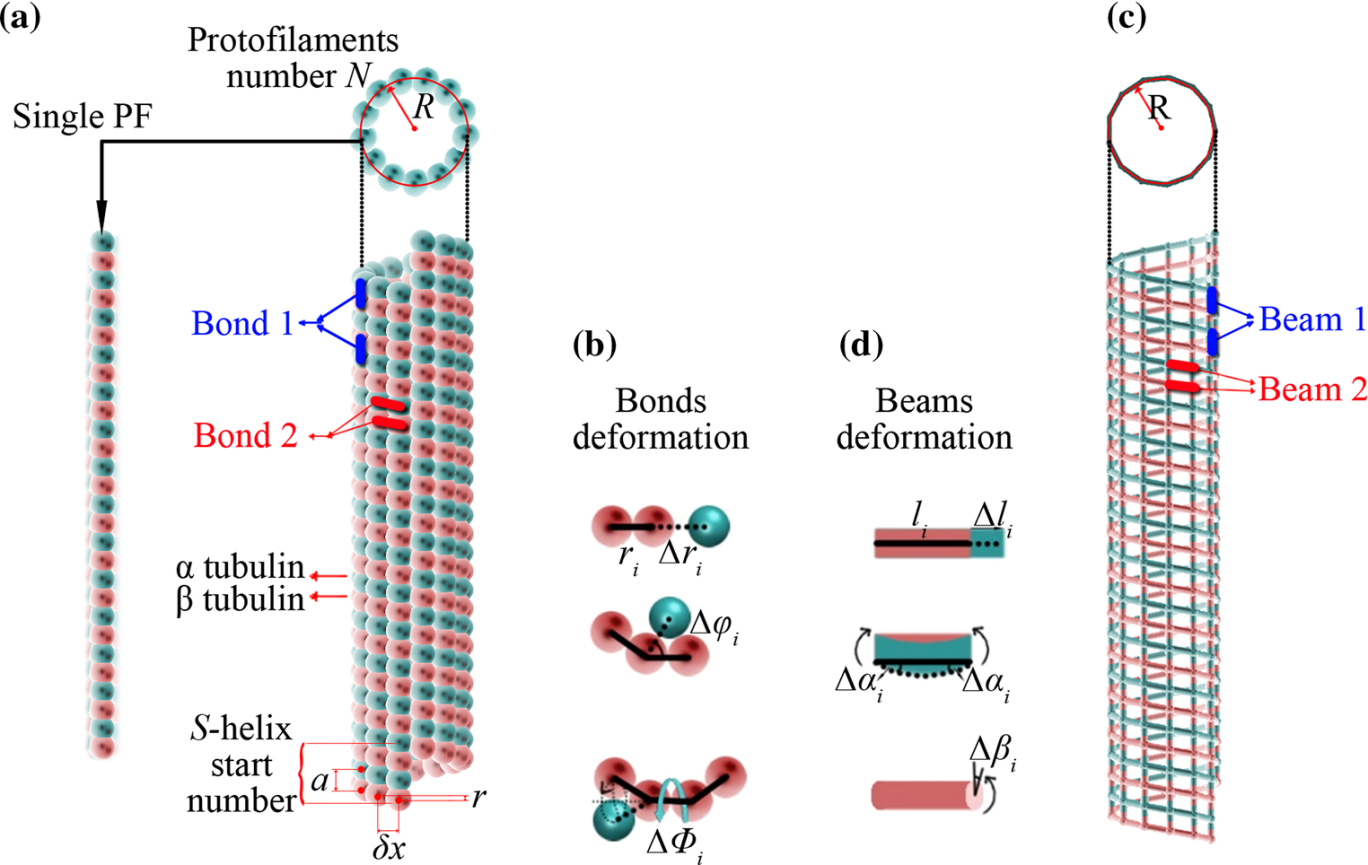
**a** A structural representation of an MT, **b** the major bond deformation considered for the MT, **c** the molecular structural model developed for the MT and **d** the deformations of the elastic beams representing bonds 1 and 2 of the MT shown in (**a**)

In 2006, Ru’s group developed an orthotropic shell model for MTs (Wang et al. 2006a, b) and later compared it with the EB model (Li et al. 2006). The length-dependent (*EI*)$_{\mathrm{eq}}$ was found to be a result of the extremely low *G* relative to the axial Young’s modulus (Li et al. 2006). Subsequently, Ru and his co-workers (Gu et al. 2009; Shi et al. 2008) confirmed the relevance of the Timoshenko beam (TB) model to MTs by comparing it with the shell model (Wang et al. 2006a, b). The length-dependence of (*EI*)$_\mathrm{eq }$ was also predicted by the TB model via the low *G*-induced transverse shear. In addition, a higher-order shear deformation theory was used by Tounsi et al. (2010) to understand the length-dependent (*EI*)$_{\mathrm{eq}}$ of MTs. This unique feature was also explained by Gao and Lei (2009) and Fu and Zhang (2010) via the nonlocal elasticity and the couple stress theory, respectively.

A CMM or an improved-CMM (Xiang and Liew 2011, 2012) is often chosen by researchers for a nanostructure primarily due to the similarity between their overall geometric configurations. However, the deformation mechanisms of discrete nanostructures may not be correctly reflected by that of a continuous body. This, in fact, forms a fundamental issue in nanomechanics. Specifically, the inter-*PF* sliding was observed experimentally for MTs (Chrétien et al. 1998; Chretien and Fuller 2000), which originates from the weak inter-*PF* interaction and is thought to be responsible for the length-dependent (*EI*)$_\mathrm{eq}$ (Kis et al. 2002; Pampaloni et al. 2006; Taute et al. 2008). Effort is thus required to further confirm this theory and examine the relevance of the CMMs to the inter-*PF* sliding of MTs.

The present paper aims to investigate this issue for the classical and nonlocal beam models (Fu and Zhang 2010; Gao and Lei 2009; Kasas et al. 2004; Kis et al. 2002; Li et al. 2006; Tounsi et al. 2010). In doing this, a molecular structural mechanics (MSM) model was employed to characterize the inter-*PF* sliding (Chrétien et al. 1998; Chretien and Fuller 2000; Wang et al. 2016) and compared with the beam models in studying the vibration and bending of MTs. The idea is to examine whether the effects of inter-*PF* sliding can be captured by the continuum beam and nonlocal mechanics theories. The MSM model has the proven ability to correlate MT structures to the elastic properties (Zhang and Wang 2014), mechanical behavior (Li et al. 2017; Zhang and Wang 2014, 2016) and particularly, the inter-*PF* sliding (Wang et al. 2016) of MTs. Here, the MSM models for MTs were introduced in Sect. 2. Section 3 discusses the numerical results and provides a critical analysis. The new findings are summarized in Sect. 4.

## Characterizing techniques for MTs

### MSM model for MTs

MTs of different architectures are found (Hunyadi et al. 2007), but the most common configuration is the standard 13-3 MTs (Chretien and Fuller 2000; Chretien and Wade 1991; Hyman et al. 1995). 13-3 MT structure is shown in Fig. 1a in which *N* ($$=$$13) is the number of *PF*s, *S* ($$=$$3) is the helix start number, $$\delta x$$ is the separation distance between two adjacent *PF*s, and *R* is the radius of the tube (Fig. 1a). In molecular mechanics, the total potential energy *U* of an MT comprises of the bond stretching energy $${U}_{{i}}^{r } $$, the angle bending energy $${U}_{{i}}^{\varphi } $$ and the dihedral angle torsional potential energy $${U}_{{i}}^{\tau } $$ (Fig. 1b).

1 $$\begin{aligned} {U}_{\mathrm{bonds}} =\sum \limits _{{i=1,2}} \left( \sum {U}_{{i}}^{r} {+}\sum {U}_{{i}}^{\varphi } +\sum {U}_{{i}}^{\tau } \right) \end{aligned}$$

The subscripts 1 and 2 denote the intra-*PF* $\upalpha \upbeta$ bonds (bond 1) and the inter-*PF* $\upalpha \upalpha$ ($\upbeta \upbeta$) bonds (bond 2) (Fig. 1a), respectively. It is noted in the molecular dynamics simulations (Enemark et al. 2008; Ji and Feng 2011) that the difference between the inter-*PF* $\upalpha \upalpha$ and $\upbeta \upbeta$ bonds is relatively small and can be safely neglected in modeling the mechanics of MTs. Specifically, it was shown (Zhang and Wang 2014) that the MSM simulations based on this assumption can be efficiently used to predict Young modulus and shear modulus of MTs in agreement with existing experimental data and theoretical results. Thus, following the treatment in previous studies (Ji and Feng 2011), the difference between $\upalpha \upalpha$ bonds and $\upbeta \upbeta$ bonds is neglected in the present work. Here, the intra-*PF* $\upalpha \upbeta$ bonds can be modeled as elastic beam 1 and the inter-*PF* $\upalpha \upalpha$ ($\upbeta \upbeta )$ bonds are treated as beam 2 (Fig. 1c). An MT (Fig. 1a) can then be considered as a frame structure (Fig. 1c) whose potential energy is

2 $$\begin{aligned} {U}_{\mathrm{beams}} =\mathop \sum \limits _{{i=1,2}} \left( {\sum {U}_{{i}}^{A} {+}\sum {U}_{{i}}^{M} {+}\sum {U}_{{i}}^{T} } \right) \end{aligned}$$

where $${U}_{{i}}^{A} $$, $${U}_{{i}}^{M} $$ and $${U}_{{i}}^{T} $$ are the strain energies in a beam due to tension, bending and torsion (Fig. 1d). The subscripts 1 and 2 denote the strain energies of the beam 1 and 2, respectively. The equivalency between an MT and its frame structure can be established when the energies in Eq. 1 are equal to the corresponding energies in Eq. 2. This condition yields

3 $$\begin{aligned} {Y}_{{i}} {A}_{{i}} {=k}_{{i}}^{r} {l}_{{i}} {,Y}_{{i}} {I}_{{i}} {=k}_{{i}}^{\varphi } {l}_{{i}} {,S}_{{i}} {J}_{{i}} {=k}_{{i}}^{\tau } {l}_{{i}} {,(i=1,2)} \end{aligned}$$

where $${Y}_{{i}} {A}_{{i}} $$, $${Y}_{{i}} {I}_{{i}} $$ and $${S}_{{i}} {J}_{{i}} $$ are the extensional, bending and torsional stiffnesses of elastic beam *i*, respectively. $${k}_{{i}}^{r} $$, $${k}_{{i}}^{\varphi }$$ and $${k}_{{i}}^{\tau } $$ are the force constants for bond stretching/compression, angle bending and torsion of MTs. $${l}_{{i}}$$ is the length of the equivalent beam *i*. Once the force constants are obtained from experiments or atomistic simulations, the beam stiffness can be obtained based on Eq. 3.

The vibration equation of the above frame structure is as follows (Li and Chou 2004; Tedesco et al. 1999)

4 $$\begin{aligned} \mathbf{M}\ddot{\varvec{\chi }}+ \mathbf{K} {{\varvec{\chi }}}={\varvec{0}} \end{aligned}$$

where $\mathbf{M}$ denotes the global mass matrices, $\mathbf{K}$ denotes the stiffness matrices, $\ddot{{\varvec{\chi }}}$ denotes the acceleration vector, and ${\varvec{\chi }}$ denotes the nodal displacement vector. For the details of $\mathbf{M}$ and $\mathbf{K}$ readers may refer to Refs. Li and Chou (2004), Tedesco et al. (1999) and Zhang and Wang (2014). The vibration modes and frequency ${f=\omega /2\pi }$ can be obtained by solving the eigenvalue problem below (Zhang and Wang 2016) via the block Lanczos algorithm.

5 $$\begin{aligned} \left( {\mathbf{K}-\omega ^{2}{} \mathbf{M}} \right) {\varvec{\chi }}=\mathbf{0} \end{aligned}$$

For the static deformation of an MT, the nodal displacements can be calculated for the frame structures of MTs via the stiffness matrix method based on the following equation

6 $$\begin{aligned} \mathbf{Ku}=\mathbf{F} \end{aligned}$$

where $\mathbf{u}$ is the global nodal displacements and $\mathbf{F}$ is the nodal forces acting on the boundary of an MT. Solving Eq. 6 gives the nodal displacements of the individual nodes and thus the deformation of MTs. This MSM technique was efficiently used in characterizing the elastic properties (Zhang and Wang 2014), buckling behavior (Zhang and Meguid 2014) and free vibration of MTs (Zhang and Wang 2016). It is found to be in good agreement with experiments and other simulations (Zhang and Wang 2014), and able to reflect the effect of the inter-*PF* sliding on MT deformation (Wang et al. 2016).

In Deriu et al. (2007), and Ji and Feng (2011), MDSs were performed to measure the force constants for MTs., ${k}_{1}^{r}=3\hbox { nN/nm}$, ${k}_{1}^{\varphi } =2\hbox { nN}\,\hbox {nm}$ and ${k}_{1}^{\tau } =0.04\hbox { nN}\,\hbox { nm}$ were obtained for the intra-*PF* $\upalpha \upbeta$ bonds, and ${k}_{2}^{r} =14\hbox { nN/nm}$, ${k}_{2}^{\varphi } =8.5\hbox { nN}\,\hbox { nm}$ and ${k}_{2}^{\tau }=0.17\hbox { nN}\,\hbox {nm}$ were calculated for the inter-*PF* $\upalpha \upalpha$ ($\upbeta \upbeta$) bonds. However, in the literature (Deriu et al. 2010; Kis et al. 2002; Li et al. 2006; Sept and MacKintosh 2010; Tuszyński et al. 2005) large discrepancy (six orders of magnitude different) is found in measuring the shear modulus *G* that is primarily determined by the inter-*PF* bonds (Pampaloni et al. 2006; Wang et al. 2016). Accordingly, in this study while the above intra-*PF* force constants were used, those of the inter-*PF* bonds considered vary in a wide range, i.e., ${k}_{2}^{r} =14\hbox { QnN/nm}$, ${k}_{2}^{\varphi } =8.5{\hbox { QnN}}\,{\hbox {nm}}$ and ${k}_{2}^{\tau } =0.17{\hbox { QnN}}\,{\hbox {nm}}$, where the coefficient *Q* ranges from $10^{-4}$ to $10^{2 }$ and alters the effect of the inter-*PF* sliding (Wang et al. 2016). These *Q* values were selected as they can return a range of shear modulus *G* in accordance with the values reported in the literature. Also, it should be pointed out here that the present MSM technique is applicable only for small deformation of MTs. No matter what *Q* value is considered, this condition can be satisfied by considering a relatively low external load or small vibration amplitude.

### Shear modulus and bending stiffness measurement

**Fig. 2:**
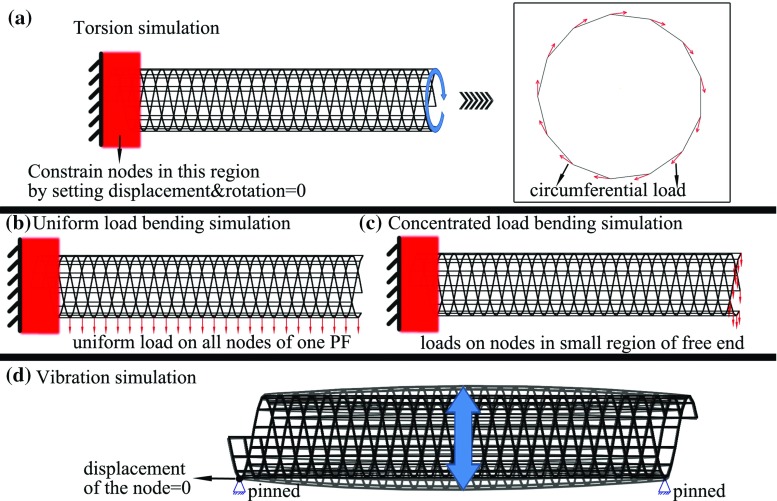
Experimental setup in the MSM simulations for **a** torsion, **b** the bending of a cantilevered MT subject to a distributed force, **c** the bending of a cantilevered MT due to a concentrated load on the free end and **d** the vibration of a simply supported MT

In this study, the MSM simulations were performed to measure the mechanical properties of the 13-3MTs. The boundary conditions and loading conditions considered in the MSM simulations were introduced below and are illustrated in detail in Fig. 2.

As shown in Fig. 2a, torsional deformation was obtained for the cantilevered MT by applying circumferential force $$F_{c}$$ on each node of the free end. The other end is fixed by imposing zero degree of freedom on the nodes very close to the fixed end (their axial distance to the end is less than 3*c* where *c* is subunit repeat along *PF*s). The shear modulus *G* can then be calculated by $$F_c RL/\left( {\gamma J_0 } \right) $$, where *L* is the unconstrained length of the MT, $$\gamma $$ is the torsional angle that is measured in the MSM simulations and $$J_0 $$ is the polar moment of inertia ($$J_0 =\left( {\pi /32} \right) [(2R+t)^{4}-\left( {2R-t)^{4}} \right] $$ and the effective thickness of MTs *t* = 2.8 nm Deriu et al. 2010). In general, anisotropic MTs may have different shear moduli in circumferential (torsional) and axial directions. However, the more detailed study based on the present MSM model (not included in the paper) showed that the two shear moduli exhibit the similar trend to change with *Q* and their values associated with a given *Q* are quite close to each other. Thus in the present study, the circumferential shear modulus *G* obtained in the torsion test was used to represent the axial shear modulus and employed in the beam models.

In addition, bending of cantilevered 13-3MTs was achieved under two loading conditions. First a uniformly distributed transverse force $q_{0}$ (N/m) is achieved on the MT by applying a transverse force $q_{n}= q_{0}L/N_{u-\mathrm{nodes}}$ on each node of the bottom *PF* (Fig. 2b). Here, $N_{u-\mathrm{nodes}}$ is the number of the loaded nodes. The transverse deflection $w_\mathrm{max}$ of the free end and the distributed force $q_{0 }$ can be measured in the MSM simulations. Thus, (*EI*)$_{\mathrm{eq}}$ of the MT can be calculated based on the EB (Eq. 7) and TB (Eq. 8) theories, respectively (Reddy and Pang 2008).

7 $$\begin{aligned} w_\mathrm{max}= & {} \frac{q_0 L^{4}}{8\left( {EI} \right) _\mathrm{eq} } \end{aligned}$$

8 $$\begin{aligned} w_\mathrm{max}= & {} \frac{q_0 L^{4}}{8\left( {EI} \right) _\mathrm{eq} }\left( {1+\frac{4\left( {EI} \right) _\mathrm{eq} }{GAK_s L^{2}}} \right) \end{aligned}$$

Here, *A* is the area of cross section; $$K_{s} = 0.72$$ is the shear correction coefficient (Deriu et al. 2010; Pampaloni et al. 2006; Zhang and Wang 2014). Alternatively, a concentrated transverse force *f* on the free end can be generated by applying a force $$f_{n}=f $$/$$N_{c-\mathrm{nodes}}$$ on the nodes whose axial distance to the free end is less than 3c (Fig. 2c). (*EI*)$$_{\mathrm{eq}}$$ of the MT can be obtained via the *EB* (Eq. 9) and *TB* (Eq. 10), respectively (Arash and Wang 2012).

9 $$\begin{aligned} w_\mathrm{max}= & {} \frac{fL^{3}}{3\left( {EI} \right) _\mathrm{eq} } \end{aligned}$$

10 $$\begin{aligned} w_\mathrm{max}= & {} \frac{fL^{3}}{3\left( {EI} \right) _\mathrm{eq} }+\frac{fL}{GAK_s } \end{aligned}$$

Additionally, simulations were performed for the transverse vibration of the simply supported MTs. The end conditions were achieved by fixing one node on each end of the MT (Fig. 2d). Here, the angular vibration frequency $\omega$ can be measured in MSM simulations and (*EI*)$_{\mathrm{eq}}$ can be obtained based on EB (Eq. 11) and TB (Eq. 12) theories, respectively (Reddy 2007; Reddy and Pang 2008).

11 $$\begin{aligned} \omega= & {} \left( {\frac{n\pi }{L}} \right) ^{2}\left( {\frac{\left( {EI} \right) _\mathrm{eq} }{m_0 }} \right) ^{1/2} \end{aligned}$$

12 $$\begin{aligned} \omega= & {} \left( {\frac{n\pi }{L}} \right) ^{2}\left( {\frac{\left( {EI} \right) _\mathrm{eq} }{m_0 \left( {1+\frac{n^{2}\pi ^{2}\left( {EI} \right) _\mathrm{eq} }{GAK_s L^{2}}} \right) }} \right) ^{1/2} \end{aligned}$$

Here, it is noticed that the *Q*-dependence of (*EI*)$_{\mathrm{eq }}$ can be obtained via *G*(*Q*), $w_\mathrm{max}(Q)$ and $\omega$(*Q*) in Eqs. (7–12).

### Nonlocal coefficient measurement

When the size of a structure miniaturizes across the length scale, one would see the changes in the constitutive relations of the material in the structure. For a bulk material, the stresses of a reference point are only a function of the strains at the same point. However, for a nanoscale material, the stresses of a reference point may be determined by the strains of all points in the domain occupied by the nanomaterial (Eringen 1976, 1983). Previously, effort was made to study the bending and vibration behavior of MTs based on nonlocal theory (Civalek and Akgöz 2010; Civalek and Demir 2011). In particular, the length-dependence of (*EI*)$_{\mathrm{eq}}$ achieved for MTs was interpreted based on the nonlocal beam models developed by incorporating the nonlocal constitutive relations into the classical beam theories (Gao and Lei 2009). For a cantilevered MT the bending deflection $w_\mathrm{max}$ of the free end is obtained below based on the nonlocal EB and TB models when a uniformly distributed force $q_{0}$ is applied (Reddy 2007; Reddy and Pang 2008)

13 $$\begin{aligned} w_\mathrm{max}= & {} \frac{q_0 L^{4}}{8\left( {EI} \right) _\mathrm{eq} }\left( {1-\frac{4(e_0 a)^{2}}{L^{2}}} \right) \end{aligned}$$

14 $$\begin{aligned} w_\mathrm{max}= & {} \frac{q_0 L^{4}}{8\left( {EI} \right) _\mathrm{eq} }\left( {1+\frac{4\left( {EI} \right) _\mathrm{eq} }{GAK_s L^{2}}-\frac{4(e_0 a)^{2}}{L^{2}}} \right) \end{aligned}$$

In addition, the angular vibration frequency $\omega$ of the simply supported MT given by the nonlocal EB and TB models, respectively, is shown below (Reddy 2007; Reddy and Pang 2008)

15 $$\begin{aligned} \omega= & {} \left( {\frac{n\pi }{L}} \right) ^{2}\left( {\frac{\left( {EI} \right) _\mathrm{eq} }{m_0 \left( {1+\frac{n^{2}\pi ^{2}(e_0 a)^{2}}{L^{2}}} \right) }} \right) ^{1/2} \end{aligned}$$

16 $$\begin{aligned} \omega= & {} \left( {\frac{n\pi }{L}} \right) ^{2}\left( {\frac{\left( {EI} \right) _\mathrm{eq} }{m_0 \left( {1+\frac{n^{2}\pi ^{2}\left( {EI} \right) _\mathrm{eq} }{GAK_s L^{2}}} \right) \left( {1+\frac{n^{2}\pi ^{2}(e_0 a)^{2}}{L^{2}}} \right) }} \right) ^{1/2}\nonumber \\ \end{aligned}$$

In Eqs. (13–16), the nonlocal effect is characterized by the coefficients $e_{0}a$, where $e_{0}$ is considered as a material constant that can be determined in experiments or the atomistic simulations, and *a* is an internal characteristics length, e.g., lattice parameter, granular size or the distance between C–C bonds for CNTs (Gao and Lei 2009). The values of $e_{0}a$ (*Q*) can be calculated by using Eqs. (13–16) once $w_\mathrm{max}(Q)$, $\omega$(*Q*) and *G*(*Q*) are determined in the MSM simulations. Here, the effort is made to explain the effect of the inter-$\textit{PF}$ sliding by the nonlocal effect, i.e., $e_{0}a$. Thus, in this study the constant (*EI*)$_{\mathrm{eq}}$ obtained when there is no significant inter-*PF* sliding, i.e., $Q>$ 1, is assumed for the MTs.

## Result and discussion

As mentioned in Sect. 1, different continuum mechanics theories are used to investigate the deformation of MTs. The length-dependent (*EI*)$_{\mathrm{eq}}$ was achieved and thought to be a result of the shear deformation or the nonlocal constitutive relations of MTs. Herein, an attempt was made to examine whether those effects proposed in the framework of the continuum mechanics theory are able to correctly reflect the deformation mechanisms of discrete MT structures.

**Fig. 3:**
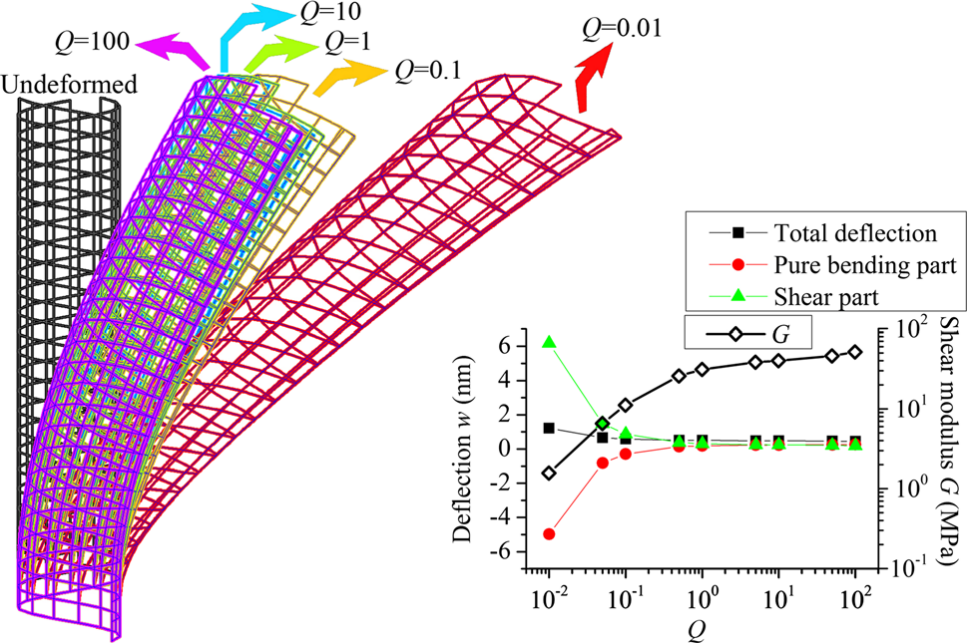
The initial position of an undeformed 13-3MT and the final position of the bent MT structures with $Q = 0.01$, 0.1, 1, 10 and 100, respectively. The illustrated displacements in the snapshots were enlarged 100-fold to reveal the differences. The inset shows the *Q*-dependence of the shear modulus *G* (diamonds), the total bending deflection of the free end (squares), the deflection due to pure bending (circles) and the one resulting from the shear deformation (triangles). The deflections were given by the TB model by using the values of *G* shown in the inset

### Inter-PF sliding of bent MTs

In this section, we investigated the effect of the inter-*PF* sliding on the bending deformation of MTs. To this end, we bent a cantilevered 13-3MT by applying a concentrated force on the free end. Here, the effect of the possible inter-*PF* sliding was altered intentionally by varying the stiffness of the inter-*PF* bonds in a broad range, i.e., the coefficient *Q* changes from $10^{-2}$ to $10^{2}$. The snapshots of the initial position (the undeformed configuration) and the final position (the bent configuration with the maximum transverse deflection) are shown in Fig. 3 for the MTs with *Q* equal to $10^{2}$, $10^{1}$, $10^{0}$, $10^{-1}$ and $10^{-2}$, respectively. The illustrated displacements in the snapshots were enlarged 100-fold to reveal the differences. The transverse deflection of the free end is found to increase when *Q* decreases or the inter-*PF* bonds become softer. However, it rises only slightly when *Q* declines from $10^{2}$ to $10^{0}$, i.e., the inter-*PF* bonds are relatively stiff. The growth becomes more significant at $Q= 10^{-1}$ and turns out to be large as *Q* reaches $10^{-2}$ or the inter-*PF* bonds become very soft.

In the meantime, we calculated the shear modulus *G* introduced for MTs in Zhang and Wang (2014). The *Q*-dependency of *G* was plotted in the inset of Fig. 3 where *G* decreases with decreasing *Q*. Specifically, consistent with the above deflection change, *G* varies only by a few times when *Q* falls in the range of [$10^{0},10^{2}$]. It, however, drops abruptly by one to two orders of magnitude when *Q*declines from $10^{0}$ to $10^{-2}$. Thus, the stiffness of the inter-*PF* bond stiffness can be approximately measured by the shear modulus *G* quantifying the shear deformation resistance of MTs.

In addition, it was also seen from Fig. 3 that at $Q=10^{2}$, i.e., the inter-*PF* bond stiffness or the shear modulus *G* is relatively high, the MT bends like a EB with the central axis (or the neutral axis) nearly perpendicular to the cross sections. This situation remains nearly unchanged as *Q* reduces from $10^{2}$ to $10^{0}$ and the transverse deflection grows slightly. In contrast, when *Q* decreases to $10^{-2}$, i.e., the inter-*PF* bond stiffness or *G* is one to two orders of magnitude lower, the inter-*PF* sliding or shear deformation can be clearly observed for the MT where the central axis is no longer perpendicular to the cross section. It follows that at $Q>10^{0}$, the small transverse deflection in Fig. 3 is primarily a result of the pure bending of the MT. By contrast, at $Q=10^{-2}$ the deflection increases greatly due to the inter-*PF* sliding or the shear deformation of the MT.

Based on the above MSM simulations it can be concluded that the soft inter-*PF* bond will lead to the large inter-*PF* sliding or the shear deformation, and thus additional (or greater) transverse deflection of MT structures. The stiffness of the inter-*PF* bonds or the resistance to shear deformation of MTs is measured by the shear modulus *G* that can be obtained in the MSM simulations. This theory is qualitatively similar to the concepts of the proposed CMMs (Gu et al. 2009; Kis et al. 2002; Li et al. 2006; Pampaloni et al. 2006; Shi et al. 2008; Tounsi et al. 2010; Wang et al. 2006a, b) where the shear deformation is considered for MTs. For example, Eq. 10 obtained based on the TB model gives the transverse deflections due to pure bending $\frac{fL^{3}}{3\left( {EI} \right) _\mathrm{eq} }$ and the shear deformation $\frac{fL}{GAK_s }$, respectively. Thus, Eq. 10 was employed to quantify the MT deflections due to the pure bending and shear deformation (or the inter-*PF* sliding). The results were also plotted in the inset of Fig. 3 where at $Q<10^{0}$, the shear deflection (solid triangles) given by the TB model (*G* is measured by the MSM model) is even larger than the total deflection (solid squares) observed in the MSM simulations. This finally leads to an unacceptable negative bending deflection (solid circles) or a negative bending stiffness of the MT. The results suggested that though the TB model is generally in qualitative agreement with the MSM simulations, it may overestimate the effect of the equivalent shear deformation or the inter-*PF* sliding in some particular cases. This situation thus necessitates a more comprehensive investigation on the relevance of the classical beam models to the mechanical deformations of MTs.

### Classical beam models for MTs

**Fig. 4:**
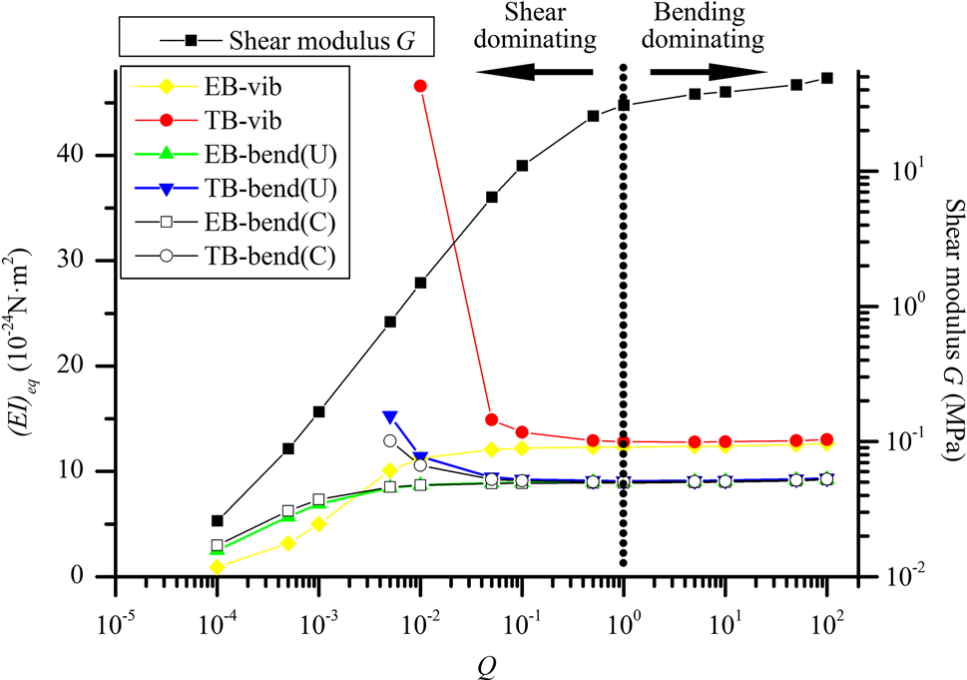
The *Q*-dependency of the shear modulus *G* (squares) obtained in the MSM simulations and that of (*EI*)$_{\mathrm{eq}}$ calculated for MT structures with $L/D=40$. (*EI*)$_{\mathrm{eq}}$ obtained for vibrating MTs based on the EB and TB models are represented by diamonds and circles, (*EI*)$_{\mathrm{eq}}$ of bent MTs under a uniform load given by the EB and TB models are denoted by triangles and upside-down triangles, and the ones for bent MTs subject to a concentrated load achieved by using the EB and TB models are represented by squares and circles, respectively

In the previous section, the bending of 13-3 MT was studied based on the MSM model and the classical beam theories. The inter-*PF* sliding of MTs was identified as the physical origin of the shear deformation considered in the TB model for MTs. In this section, an investigation was carried out to further examine the relevance of the beam models to the mechanics of MTs. To this end, the *Q*-dependency of (*EI*)$_{\mathrm{eq}}$ was calculated in Fig. 4 by fitting the EB and TB models to the MSM simulations on the vibration or bending of MTs. Herein, 13-3 MT structures were considered where the length *L* is fixed at $\sim$0.85 $\upmu$m, i.e., the length-to-diameter ratio *L*/$D = 40$, and *Q* varies between $10^{-4}$ and $10^{2}$. The shear modulus *G* is also shown in Fig. 4 to understand the trend of (*EI*)$_{\mathrm{eq}}$.

In the range $10^{0 }<Q<10^{2}$, *G* in Fig. 4 falls in the range of [30.8, 48.8 MPa] where, as shown in Sect. 3.1, the effect of the inter-*PF* sliding is very small or negligible. Thus, (*EI*)$_{\mathrm{eq}}$ obtained based on the frequency of MT vibration is nearly a constant around 13 $\times$ 10$^{-24}$ Nm$^{2}$. The difference between the EB (solid diamonds) and TB (solid circles) due to shear modulus *G* was found to be small showing that the MT vibrates like an EB where the effect of the shear deformation or the inter-*PF* sliding is trivial.

In the range $10^{-4 }<Q<10^{0}$, *G* decreases greatly from around 30.8 to 0.026 MPa as *Q* declines. The *G*-variation of three orders of magnitude is found to be in the same range of *G* values reported in the literature (Deriu et al. 2010; Kis et al. 2002; Li et al. 2006; Sept and MacKintosh 2010; Tuszyński et al. 2005). In this process when the inter-*PF* bonds become softer, the effect of the inter-*PF* sliding turns out to be more significant leading to more compliant MT structures and thus, a lower frequency. Accordingly, in Fig. 4, (*EI*)$_{\mathrm{eq}}$ given by the EB model (solid diamonds) is found to decrease with decreasing *Q*. In other words, the EB model interprets the lower frequency due to the enhanced effect of the inter-*PF* sliding (or increased shear deformation due to lower *G*) in terms of the decreasing (*EI*)$_{\mathrm{eq}}$. In other words, the EB model is unable to reflect the real deformation mechanisms of the discrete MT structure with softer inter-*PF* bonds.

In contrast to the EB model, (*EI*)$_{\mathrm{eq}}$ obtained based on the TB model (solid circles) climbs up when *Q* drops from $10^{0}$ to $10^{-4}$. As shown in Sect. 3.1, the TB model is considered to be more relevant to MTs as the shear deformation or *G* of the TB can quantitatively explain the effect of the inter-*PF* sliding (Chrétien et al. 1998; Chretien and Fuller 2000). Nevertheless, the *Q*-dependence of (*EI*)$_{{ eq}}$ (solid circles) found in Fig. 4 is not true for the MTs. In fact, the MSM simulations (the results are not shown here) showed that the axial Young’s modulus *E* ($\approx$0.8 GPa) and the second moment of inertia *I* are not sensitive to the change in the inter-*PF* bond stiffness or the coefficient *Q*. In other words, (*EI*)$_{\mathrm{eq}}$ defined as the product of *E* and *I* should be nearly a constant independent of *Q*. Thus, the predicted *Q*-dependence of (*EI*)$_{\mathrm{eq}}$ suggested again that the TB model overpredicts the softening effect of the inter-*PF* sliding on MT structures. As a result, (*EI*)$_{\mathrm{eq}}$ of the TB model has to be raised to counterbalance the overestimated effect of the inter-*PF* sliding (or the shear deformation) and keep the obtained frequencies the same as those of the MSM model. This observation is consistent with the one for MT bending in Sect. 3.1.

**Fig. 5:**
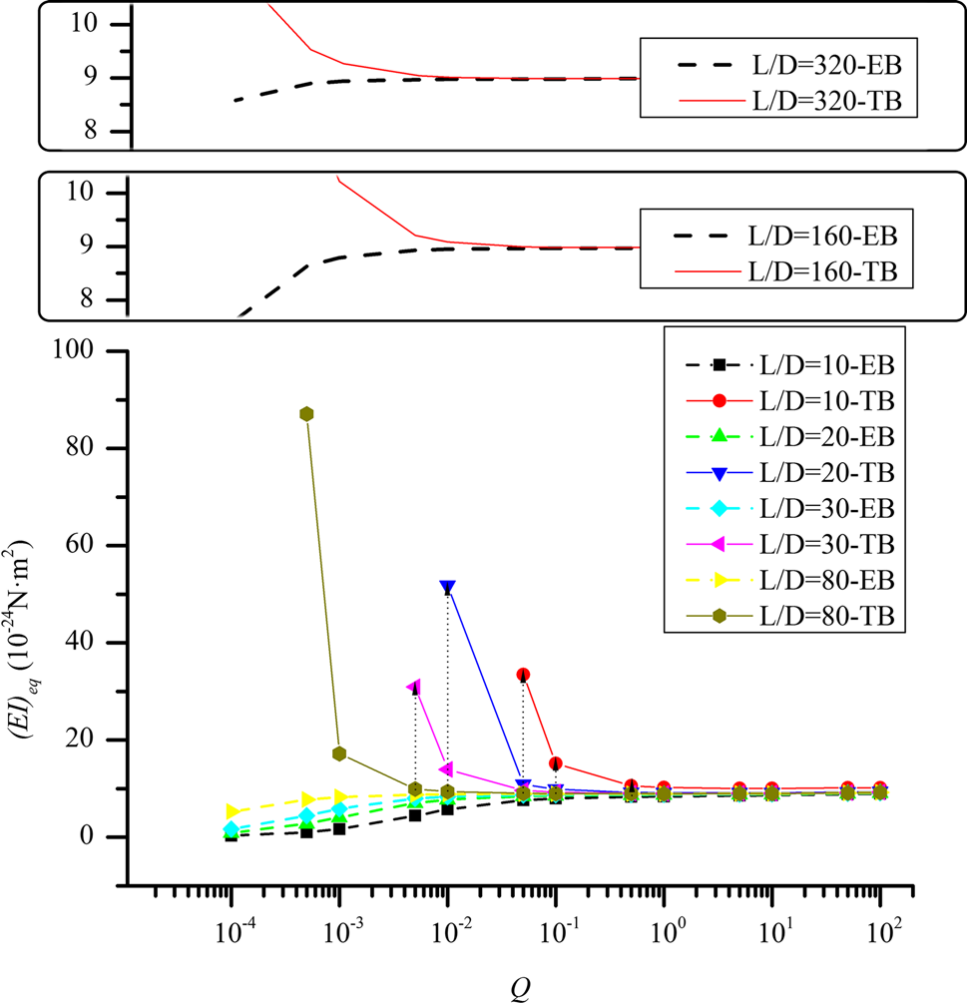
The *Q*-dependency of (*EI*)$_{eq }$ obtained for bent MT structures which are subject to a distributed transverse load and possess *L*/*D* rising from 10 to 320

The *Q*-dependence of (*EI*)$_{\mathrm{eq}}$ was also achieved in Fig. 4 via the bending tests in the MSM simulations. The results for the MTs subject to a distributed force (Fig. 2b) and forces on the free end (Fig. 2c) were nearly the same and qualitatively similar to those obtained via the vibration of the simply supported MTs (Fig. 2d). The major difference in these two cases is that, at $10^{0}<Q<10^{2}$ the constant (*EI*)$_{\mathrm{eq}}\approx$ 9 $\times 10^{-24}$ Nm$^{2}$ obtained in MT bending is lower than $\sim$13 $\times$10$^{-24}$ Nm$^{2}$ measured in MT vibration. The discrepancy can be partially attributed to the different boundary conditions considered in the bending and vibration of the MTs. These results thus support the conclusions drawn above based on the *Q*-dependence of (*EI*)$_{\mathrm{eq}}$ achieved via MT vibration.

Furthermore, in Fig. 5 the *Q*-dependency of (*EI*)$_\mathrm{eq }$ was calculated for the uniformly loaded bending of 13-3 MT structures whose aspect ratio *L*/*D* rises from 10 to 80. The results were analogous to what was observed in Fig. 4, i.e., when *Q* is relatively large and the effect of the inter-*PF* sliding is small, both the EB and TB models give nearly the same (*EI*)$_\mathrm{eq}\approx 9\times 10^{-24}$ Nm$^{2}$ independent of *Q* and the aspect ratio *L*/*D*. In this case the (*EI*)$_\mathrm{eq }$ curves given by the two beam models nearly coincide with each other. However, when *Q* decreases the (*EI*)$_{\mathrm{eq}}$ curves of the two beam models bifurcate at a critical value $Q_\mathrm{cr}$ and then show the reversed trend of (*EI*)$_{\mathrm{eq}}$. The values of $Q_\mathrm{cr}$ decrease from $1\times 10^{0}$ to $1\times 10^{-1}$ and $1\times 10^{-2}$ when the aspect ratio rises from 10 to 30 and 80. Further increasing the aspect ratio to 160 and 320 leads to less pronounced decreasing trend of $Q_\mathrm{cr}$. These situations considered in Fig. 5 are close to the MTs found in cells, which are usually 1–10 $\upmu$m long (Pampaloni et al. 2006) or aspect ratio 40–400. It is found in the figure that $Q_\mathrm{cr}$, i.e., the maximum *Q* value associated with the substantial inter-protofilament sliding, varies between $5\times 10^{-2}$ and $1\times 10^{-3}$. The corresponding *G* values, as shown in Fig. 3, lie in the range of [0.163–6.421 MPa]. These results suggested that the upper limit of the shear modulus of MTs should be at the order of 1 MPa, which is close to the shear modulus 1.4 MPa measured in Kis et al. (2002). The higher shear modulus *G* is unlikely as it would prevent the inter-protofilament sliding that has already been observed for MTs in the experiments (Chrétien et al. 1998; Chretien and Fuller 2000; Dye et al. 1993).

It is clearly seen from Fig. 5 that, when $Q<Q_\mathrm{cr}$, (*EI*)$_{\mathrm{eq}}$ of the MTs exhibit substantial length-dependence (see vertical dotted lines in Fig. 5), whereas when $Q>Q_\mathrm{cr}$, (*EI*)$_{\mathrm{eq}}$ remains a constant without significant *Q*- and length-dependence. These results further confirmed that the inter-*PF* sliding resulting from the soft inter-*PF* bonds is the physical origin of the length-dependence of (*EI*)$_{\mathrm{eq}}$ obtained based on the classical beam theories. In other words, the MT structures behave like a EB (or TB) with a constant (*EI*)$_{\mathrm{eq}}$ when their inter-*PF* bonds are stiff and the effect of the inter-*PF* sliding is very small or negligible. However, as far as the soft inter-*PF* bonds are concerned or the softening effect of the inter-*PF* sliding becomes substantial, the length-dependence of (*EI*)$_{\mathrm{eq}}$ emerges because the EB model is unable to account for the effect of the inter-*PF* sliding or shear deformation and the TB model overestimates its softening effect.

Here, it is clearly seen from the above analyses that the agreement of a CMM with discrete simulations can be achieved by using the elastic modulus or structural stiffnesses obtained via curve fitting. The identical numerical values, however, do not necessarily confirm the relevance of the CMM to the nanostructure as the curve fitting results may not correctly imitate the physical mechanisms of MT deformations. This is simply due to the distinct deformation mechanisms between a discrete nanostructure and its equivalent continuous body of similar geometric configuration.

### Nonlocal beam models for MTs

In this section, the nonlocal effect characterized by the nonlocal coefficient $e_{0}a$ was employed to quantify the influence of the inter-*PF* sliding on MT structures, which, as shown above, can also be measured by the equivalent shear modulus *G* or the coefficient *Q*. The goal is to examine the relevance of the nonlocal theories to the effect of the inter-*PF* sliding, a unique deformation mechanism of MT structures.

**Fig. 6:**
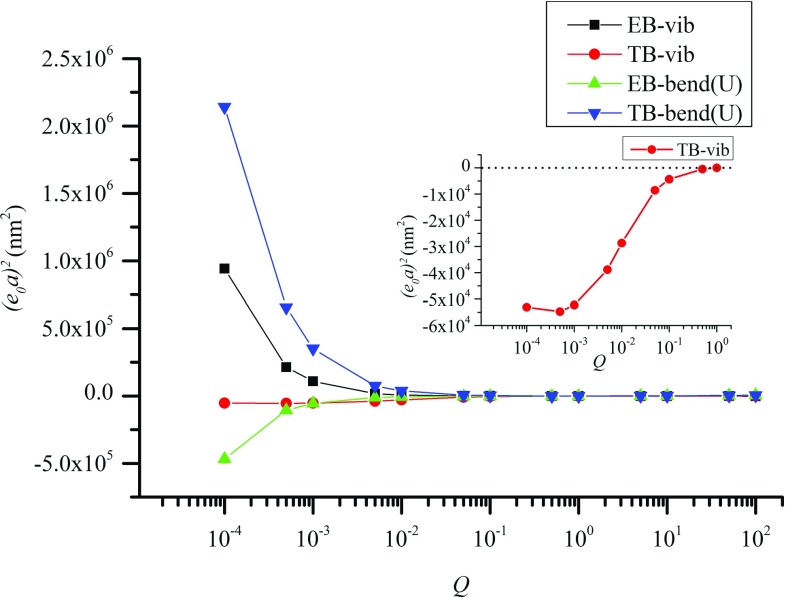
The *Q*-dependence of ($$e_{0}a)^{2}$$ calculated for 13-3MT structures. The data obtained for the vibrating MT structures based on the nonlocal EB and TB models are represented by squares and circles, respectively, and those for the bent MTs by using the nonlocal EB and TB models are denoted by triangles and the upside-down triangles, respectively. Negative values of $$(e_{0}a)^{2}$$ shown in the figure indicate the situation where the nonlocal beam model is not relevant for the mechanics of MTs

In doing calculations the shear modulus *G* shown in Fig. 4 was used in the TB model, which decreases with declining *Q*. On the other hand, constant $$\left( {EI} \right) _\mathrm{eq} $$ associated with $$Q>$$ $$10^{0}$$ in Fig. 4 was used for both nonlocal EB and TB models as its value (not the curve fitting one) does not change significantly with *Q*. Here, the *Q*-dependence of ($$e_{0}a)^{2}$$ was calculated based on Eqs. 13 and 15 (the nonlocal EB theory) and Eqs. 14 and 16 (the nonlocal TB theory) in studying the bending and vibration of the MT structures, respectively. The results were plotted graphically in Fig. 6.

First let us consider the results obtained from the vibration of simply supported MTs. It was shown in Fig. 6 that ($$e_{0}a)^{2}$$ achieved based on the EB model (solid squares) decreases from 942,106 nm$$^{2}$$ to a value close to 0 when *Q* rises from $$10^{-4}$$ to $$10^{0}$$, i.e., the inter-*PF* bonds become stiffer and the effect of the inter-*PF* sliding or the nonlocal effect decreases. As expected, ($$e_{0}a)^{2}$$ finally becomes very small when *Q* is further raised from $$10^{0}$$ to $$10^{2}$$, showing very small or negligible inter-*PF* sliding or the nonlocal effect. In this case, as shown in Sects. 3.1 and 3.2 the MT structures can be approximately modeled as a classical EB. These seem to suggest that the growing effect of the inter-*PF* sliding due to softening of the inter-*PF* bonds can be adequately captured by the nonlocal EB model.

On the other hand, ($$e_{0}a)^{2}$$ given by the TB model (solid circles) showed an opposite trend in Fig. 6 where negative ($$e_{0}a)^{2}$$ is found at $$Q<$$ $$10^{0}$$ (inset) and it approaches 0 at $$Q>$$ $$10^{0}$$. The latter matches the results of the EB model. The former, however, is a trivial solution without real physical explanations. As shown in Sects. 3.1 and 3.2, the TB model accounts for the inter-*PF* sliding in terms of the shear deformation, but it overestimates its effect on MT vibration, i.e., the MT frequency given by the TB model is even lower than the one obtained in the MSM simulations. Thus, when the nonlocal effect is incorporated into the TB model, negative ($$e_{0}a)^{2}$$ is required to upshift the frequency and make it equal to the MSM value. Positive ($$e_{0}a)^{2 }$$, however, signifies the softening nonlocal effect on the simply supported MTs, which further decreases the frequency. It is thus clear that considering both the shear deformation and the nonlocal effect may not lead to a beam model more suitable for MTs than the one with only one of the two effects.

Next we considered the data in Fig. 6 collected for the bending of cantilevered MTs. In this case, ($$e_{0}a)^{2}$$ of the nonlocal EB model (solid triangles) grows with rising *Q* (Fig. 6) and remains negative at $$10^{-4 }<Q<10^{0}$$ where the softening effect of the inter-*PF *sliding is substantial. The trend of ($$e_{0}a)^{2}$$ and specifically the negative ($$e_{0}a)^{2}$$ obtained for the cantilevered MTs (solid triangles) are found to be different from those of the simply supported MTs (solid squares). The discrepancy is due to the sensitivity of the nonlocal effect on the end conditions of beams (Reddy and Pang 2008). While it exerts softening influence on the simply supported beams (e.g., a lower frequency given by Eqs. 15 and 16) it generates stiffening effect on the cantilevered ones (e.g., a smaller bending deflection given by Eqs. 13 and 14). In contrast to this, the inter-*PF* sliding always results in a more compliant MT structure with a lower vibration frequency or a larger bending deflection. The meaningless negative ($$e_{0}a)^{2 }$$ is thus a result of the reverse influence of the nonlocal constitutive relations and the inter-*PF* sliding on the cantilevered MTs. Thus, the nonlocal EB model, adequate for simply supported MTs as shown above, is found to be unsuitable for the cantilevered MT structures when the inter-*PF* sliding is substantial. The *Q*-dependence of ($$e_{0}a)^{2}$$ given by the nonlocal TB model (solid triangles) was also shown in Fig. 6 where ($$e_{0}a)^{2}$$ grows with decreasing *Q* or increasing softening effect of the inter-*PF* sliding. Here, the stiffening nonlocal effect associated with positive ($$e_{0}a)^{2}$$ is again in contradiction with the softening effect of the inter-*PF *sliding. Thus, the nonlocal beam models are unable to capture the deformation mechanisms of the cantilevered MTs with large inter-*PF* sliding.

## Conclusions

MSM simulations were performed to study the bending and vibration of 13-3 MTs. The shear modulus *G*, the bending stiffness (*EI*)$$_{\mathrm{eq}}$$ and the nonlocal coefficient $$e_{0}a $$ were measured for the MT structures based on the MSM model, CMMs and nonlocal mechanics theory. The unique features were achieved and elucidated via the shear deformation or the nonlocal constituent relations.

It is found that the inter-*PF* sliding may occur for the MT structures in transverse bending or vibration due to the soft inter-*PF * bonds whose stiffness can be measured roughly by the equivalent shear modulus *G*. When *G* is in the order of 10 MPa (Deriu et al. 2010; Sept and MacKintosh 2010), the inter-*PF* interaction is sufficiently strong to largely prevent the adjacent *PF*s from sliding relative to each other. Thus, an MT deforms as a classical EB with its central line perpendicular to the cross section and its bending stiffness (*EI*)$_{\mathrm{eq}}$ independent of the length.

Nevertheless, at 0.01 MPa $<G<$ 10 MPa (Kis et al. 2002; Li et al. 2006) the inter-*PF* bonds become much softer, which yields substantial inter-*PF* sliding, and thus more flexible MT structures with a lower vibration frequency or a larger bending deflection. In particular, *G* in the order of 1 MPa can be considered as an upper limit of the possible shear modulus of MTs. It is shown that the EB is unable to reflect this deformation mechanism. The TB model describes the inter-*PF* sliding via the shear deformation but overestimates its softening effect. These finally yield the length-dependence of (*EI*)$_{\mathrm{eq}}$ for MTs. In addition, the nonlocal beam models are unable to fully reflect the softening effect of the inter-*PF* sliding as its effect depends sensitively on the end conditions of MTs.

It is noted that the discrepancy between the MT structures and the proposed continuum mechanics theories is a result of the distinct deformation mechanisms between the discrete MT nanostructure and its equivalent continuous body.
